# A Model Independent S/W Framework for Search-Based Software Testing

**DOI:** 10.1155/2014/126348

**Published:** 2014-09-11

**Authors:** Jungsup Oh, Jongmoon Baik, Sung-Hwa Lim

**Affiliations:** ^1^Division of Information Systems, NSE Inc., Daejeon 305-700, Republic of Korea; ^2^Department of Computer Science, KAIST, Daejeon 305-701, Republic of Korea; ^3^Department of Multimedia, Namseoul University, Cheonan 331-701, Republic of Korea

## Abstract

In Model-Based Testing (MBT) area, Search-Based Software Testing (SBST) has been employed to generate test cases from the model of a system under test. However, many types of models have been used in MBT. If the type of a model has changed from one to another, all functions of a search technique must be reimplemented because the types of models are different even if the same search technique has been applied. It requires too much time and effort to implement the same algorithm over and over again. We propose a model-independent software framework for SBST, which can reduce redundant works. The framework provides a reusable common software platform to reduce time and effort. The software framework not only presents design patterns to find test cases for a target model but also reduces development time by using common functions provided in the framework. We show the effectiveness and efficiency of the proposed framework with two case studies. The framework improves the productivity by about 50% when changing the type of a model.

## 1. Introduction

In the era of machine to machine such as IoT (Internet of Things), the importance of software dependability rapidly increases because various pieces of software are embedded on each machine and are operating collaborating with each other by taking the core roles in ICT (information communication technology). To increase software dependability, effective and efficient software testing techniques based on Research on Search-Based Software Engineering (SBSE) should be deeply studied. SBSE has been increasing by 20% every year in terms of the number of published papers since the year 2000 [1]. Search-Based Software Testing (SBST) is one of the most dominant research areas in SBSE with more than 50% of papers published related to testing and debugging (Publication trend analysis data source: SBSE repository at http://crestweb.cs.ucl.ac.uk/resources/sbse_repository/). Because SBST is widely used to search useful test inputs among input domains, it is the most effective area of SBSE.

SBST can be effectively applied to Model-Based Testing (MBT) because MBT is to search useful test input data among the input domains of a target model. Previous studies on MBT have tried to generate test cases using static analyses [2, 3]. The more complex the structure of a model is, the harder the static analysis for the model is. It is very hard to generate test cases from a complex model using a static analysis. Recently, the researchers on MBT are trying to adopt dynamic SBST techniques to generate test cases. Dynamic SBST employs search-based methods for software testing by executing the target source code or model. Therefore, dynamic SBST is far less affected by the complexity of a model than static SBST because it determines test data using search-based policies [4, 5].

However, in MBT, if a target model has changed from one type to another, all functions of the used algorithm must be reimplemented for the new type of a model because of the differences of their target models even if the search technique is the same as the one used for the previous model. If dynamic SBST focuses only on the outputs produced by the execution of a target model, dynamic SBST may be applied to various models without changes. In other words, dynamic SBST can apply the same algorithm to various types of models because it is weakly dependent on the used models.

However, the dynamic SBST should be supported by a model-independent software framework. A model-independent software framework is an essential part of the implementation of an automatic test case generator in order to apply dynamic SBST to various models. The model-independent software framework has a set of basic programming interfaces, which are required to execute a target model, to implement a test case generator for state-based models. It also provides a software architecture and data structures to implement a test case generator.

There are several advantages of a model-independent software framework as the following. First, it can be applicable for different domains using the same algorithm because each domain has the de facto standard software model such as Petri-Net, UML, and SL/SF. Second, it can help to save the implementation effort to generate test cases for various models by reusing the provided APIs. Third, it can create highly reliable test case generator using the certified components in the framework.

In this paper, we propose a novel model-independent software framework, where SBSTs can be applied in MBTs. The contributions of our paper are as follows:

1. to present a model-independent software framework that can be used to implement a search-based test case generator for MBT,
2. to provide practical interfaces, standard data structure, and common functions that can help to apply a search-based software testing algorithm to various models,
3. to show the effectiveness and efficiency of the proposed software framework through two case studies.

The rest of this paper is organized as follows.  Section 2 discusses some related works, and Section 3 proposes the software framework and essential interface functions. In Section 4, we present two case studies in order to show the effectiveness and efficiency of our framework. Then, we conclude in Section 5.

## 2. Related Works

Most existing works of research on SBST have been related to structural testing. Structural testing is also called a white box testing because it builds up test data from the internal structure of a target piece of software. Although the number of studies on SBST related to Model-Based Testing has been increasing recently, many of them are still insufficient in terms of testing various types of models. In this section, we describe the related works on structural testing and Model-Based Testing using SBST.

### 2.1. Structural Testing

In general, structural testing schemes use a control flow graph (CFG) of a target program [6]. In a CFG, a node represents a statement or a set of statements which does not have a branching statement. Among the nodes, the nodes related to the decision become predicate (or branching) nodes. If the output of the previous branching node affects the execution of the present node, then we say that there is a control dependency between the two nodes [7]. A control dependency graph expresses a set of dependency information among the nodes in a target program. A control dependency graph provides very important information to generate test data and is widely used in various algorithms, which will be presented in the rest of this section.

Structural testing methods can be classified into static methods and dynamic methods. Static methods generate test data not by executing a target program but by analyzing the internal structure of a program. Symbolic execution methods and domain reduction methods are kinds of static methods. Dynamic methods generate test data by observing the outputs of a target program. Random testing, local search, goal-oriented approach, and chaining approach are kinds of dynamic methods.

Symbolic execution [8] method does not execute a target program but narrows down the scope of the symbol's feasible values by following the path which is generated using a CFG. These constraint satisfaction problems are NP-Complete [9]. Symbolic execution does not work well if there are indexes of arrays or loops. Moreover, this method hardly deals with runtime objects such as pointers and procedure calls. A few works are proposed to cope with the problem [10, 11] using heuristic methods. Boyer et al. [10] employed hill climbing algorithm, and Ramamoorthy et al. [11] adopted trial-and-error procedure. However, these works have a drawback, in which the complexity of the symbolic execution is getting rapidly higher as the number of paths increases.

Whereas symbolic execution methods try to find out the constraints for input variables, domain reduction methods find out a solution for the constraints. Actually, these methods were presented as a part of constraint-based testing [12]. Domain reduction methods also suffer from the same problems (e.g., loops, arrays, pointers, and procedure) as symbolic execution methods because the constraints used in domain reduction methods are created by symbolic execution methods. To relieve these problems, dynamic domain reduction method is proposed [13], which dynamically reduces the domain of input variables. However, it does not solve the index problem of arrays and loops.

Static methods like symbolic execution could not deal with the index of arrays, loops, and pointers because their actual values are provided only during the program runtime. However, dynamic methods overcome this problem because they execute a target program. Random testing, which is one of the simplest dynamic methods, executes a target program with random input data and observes the outputs. However, it hardly generates test cases for some conditions (e.g., finding *x* and *y* that satisfies “*x* = = *y*,” where *x* and *y* are real numbers).

In 1976, Miller and Spooner proposed a method to find out test data by executing a target program and by searching with specially chosen input data [14]. After 1990, the search method which exploits the branch distance of a Pascal program was proposed [15]. Branch distance, which is used in most local search algorithms, shows how far a proposition is from the true value. However, local search may not find the global optimum if a local optimum exists.

To overcome the local optimum problem of the local search, several studies employ simulated annealing for structural data generation [16, 17]. These studies can generate test data for specific paths or statements. These methods do not intensively exploit information about the proven successful paths not to be seized by the local optimum, which may cause another crucial problem. For example, in nested if statements, the branch distance reducing process for the inner if statement may violate the condition for the outer if statement, which has been already proven successful. The violation occurred because the method treats the branch distance of the inner if statement and that of the outer if statement separately.

Some studies employ genetic algorithms to the structural test data creation, and these studies can be classified into coverage-oriented methods and structure-oriented methods [6]. In coverage-oriented methods, the fitness function shows how much the individual covers the structure of a target program. The more the individual covers a larger part of the structure of the program, the better the individual is [18]. However, this method may not successfully find out the values for deeply nested statements or rarely occurring conditions because it chooses the individual that only covers the longest paths. To overcome this problem, a penalty policy method is proposed, in which a penalty is charged when a path that was already found is revisited [19]. However, this method can only be applied to simple programs.

Structure-oriented methods can be classified into branch distance oriented methods, control-oriented methods, and combined methods, in accordance with the type of information that the objective function uses. Branch distance oriented methods use the branch distance, which is employed in local search methods, as the objective function. Xanthakis et al. [20] applied a genetic algorithm only for the part not covered by the random search, but they could not overcome the local optimum problem. Another study uses the objective function, which is made up by the branch distance for the target branch [21]. Another study finds out the individuals that reach the branches and obtains the test data for the conditions of each branch [22]. However, these two studies are not sufficiently effective [6].

The function used in loop testing [21] is one of the most typical objective functions in control-oriented methods. In the research, the objective function uses the difference between the actual loop iteration number and the expected loop iteration number. A study uses the number of control-dependent nodes, which should be passed to reach the target in the control dependence graph, as the objective function [23]. Control-oriented methods may bring out many plateaus because they do not have distance information about the branch predicate.

In combined methods, the objective function uses both branch distance and control information. Tracey et al. [24] used the value computed by (1) for the objective function. In (1), “executed” is the number of executed branches, “dependent” is the number of branches to be executed, and “brandist” is the branch distance. However, Tracey's research also suffers from the local optimal problem [6]. Wegener et al. [25, 26] used (2) for the objective function. In (2), the branch distance is mapped between [0,1] and added to “approach level,” which is the number of control-dependent nodes to be executed:

(1) $$\begin{array}{c} f\left(X\right)=\left(\frac{\text{executed}}{\text{dependent}}\right)*\text{bran}\_\mathrm{dist}, \end{array}$$

(2) $$\begin{array}{c} f\left(X\right)=\text{approach}\_\text{level}+\text{bran}\_\mathrm{dist}. \end{array}$$

### 2.2. Model-Based Testing

For MBT, several different algorithms were proposed according to the type of models. SBST has been employed as an algorithm to generate test cases for various models. To generate a unique input-output sequence for an FSM (Finite State Machine), several SBST methods were proposed [27]. Derderian et al. [28] employed a genetic algorithm using temporal constraints to generate test data for FSM. A fitness function uses the number of temporal constraints violated by each candidate input sequence.

Windisch [29] employed simulated annealing, genetic algorithms, and particle swarm optimization to generate a continuous input signal for real-time SL/SF (Simulink/Stateflow) models. Signals are generated by the sequence of the individual signal blocks. The fitness function uses approach level and branch distance, as shown in (2).

Zhan and Clark [30] employed SBST to effectively generate test data appropriate for branches. They applied the metaheuristic search methods only for the parts that were not covered by the random search of Xanthakis et al. [20]. Zhan and Clark [31] used the simulation-based method as the SBST in a MATLAB/Simulink model. They executed the blocks in a black box style to generate test data for the blocks.

Most previous works of research tried to find out test data by static analysis of the model without execution of the model. Recently, several works of research have tried to find out test data by executing a target model. Yano et al. [5] generated test input sequences for Extended Finite State Machine (EFSM) by executing a target model. They presented the multioptimal solution, which supports high coverage and short length without the length limit of input sequence. Oh et al. [4] obtained a fitness value of the candidate input sequence that is obtained by using the information of the running path, which is given by executing a target model with a candidate input sequence. Then, Oh et al.'s method could generate the test case satisfying transition coverage for an SL/SF model without any static analysis.

However, to the best of our knowledge, there is no study about a model-independent algorithm for the various types of models. In this paper, we present a novel model-independent software framework which does not require static analysis and is appropriate for dynamic SBST. The proposed software framework may provide a good infrastructure for the easy and rapid generation of test data using SBST for the various types of models.

## 3. A Model-Independent Software Framework 

**Figure 1:**
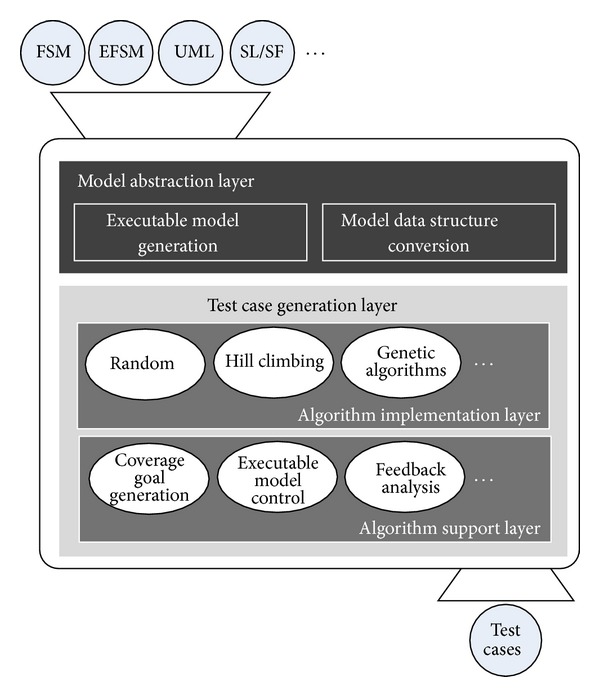
The architecture of a model-independent software framework.

The proposed framework is composed of the two different layers, a model abstraction layer and a test case generation layer. The term model used in this paper means a state-based model such as FSM, EFSM, UML, and SL/SF.  Figure 1 shows the overall architecture of the proposed software framework. The model abstraction layer processes the peculiar parts of the model, which take on roles of creating data structures or building an executable model for the test case generation layer. An executable model is the model which can be executed by the test case generation layer to select test data. The framework takes a model as an input and then generates test cases as an output. The test case generation layer takes charge in generating test cases with data structures and the executable model.

### 3.1. The Model Abstraction Layer

An executable model is the most important part of dynamic SBST because the fitness value for the test input selection is determined while running the executable model. An executable model can be generated using the source code. An executable model generation function is located in the model abstraction layer because different tools are required to generate the source code, which are the main resource of an executable model. Most modeling tools provide source code generation functions. Commercial tools for UML modeling, such as IBM Rational Rose, IBM Rational Rhapsody, and Borland Together, provide automatic source code generation tools. In FSM or EFSM, source code can be generated using a state machine compiler (SMC) (http://smc.sourceforge.net/). In an SL/SF model, source code can be generated using the Simulink coder (http://www.mathworks.com/products/simulink-coder/index.html) MATLAB package.

Before an executable model is built from a source code, interfaces to control the executable model should be merged with the source codes, which are generated using model specific tools. The interfaces are combined with the generated source code. The essential interfaces are as follows.

- Model initialization: initialize the executable model to be executable.
- Model execution: execute the executable model with the candidate input.
- Model termination: terminate the executable model to finalize the model.
- Candidate test input setting: set candidate input to the executable model.
- Execution results acquisition: get the execution results from the executable model.
- Model status acquisition: get the current active state lists and the values of variables of the executable model.

An executable model is generated by combining the predefined interfaces and a source code automatically generated from a model. An executable model appears as a dynamic-link library. The same algorithm implemented in the test case generation layer can be used to generate test cases for the various types of models because the executable models are loaded dynamically and have the same interfaces.

The final job performed in the model abstraction layer is to transform the model into a data structure which can be handled by the test case generator. Because the focus of this paper is dedicated to the state-based model, every model has charts, states, transitions, and predicates of the transition. The data structure is generated from an input model. The model abstraction layer saves all static features of an input model in the data structure. The model data structure is the most suitable data structure for SL/SF model because SL/SF model is the most complicated state model among FSM, EFSM, UML, and SL/SF. For the simpler models such as FSM, the proposed framework allows some data structures to be kept empty.

### 3.2. The Test Case Generation Layer

In the test case generation layer, SBST algorithms can be implemented and applied regardless of the type of models. The test case generation layer is divided into an algorithm implementation layer and an algorithm support layer, as shown in Figure 1. In the algorithm implementation layer, SBST algorithms, such as random, hill climbing, and genetic algorithm, are practically implemented. Supporting functions, such as coverage goal generation, executable model control, and feedback analysis, are located in the algorithm support layer. The functions in the test case generation layer are independent of the type of models and the kinds of search algorithms.

We briefly explain the basic idea of a test case generation algorithm for the state-based model, which is employed in the framework. A base node is defined as a snapshot of the state-based model. In the state-based model, the state and variables are changed according to input data. Therefore, the active state and the values of the variables of a specific point of time can be obtained by generating a snapshot of the point. To recover from the snapshot efficiently, the input sequence from the initial state to the snapshot should be stored and used. A base node is a snapshot of the model containing an active state list, values of variables, and the input sequence.

To cover the transition coverage, which is one of the most important goals, the input sequence should be found to satisfy each transition of a target model. The search process will be quite simple if we begin from the source state of the target transition. Therefore, an SBST algorithm can be modified as follows. First, find out the base node, in which its active state list has the source state of the target transition. Second, find out the test input, by which the target transition can be covered from the base node. With the above modification of the algorithm, we can implement the algorithm which can be easily applied in the algorithm implementation layer.

#### 3.2.1. The Algorithm Implementation Layer

In the algorithm implementation layer, various SBST algorithms can be employed. Many SBST algorithms select the test cases among several candidate test inputs according to the fitness of the candidate inputs. Therefore, the fitness function for selecting the test inputs among the candidates is defined in the algorithm implementation layer. Various dynamic SBST algorithms can be embedded in this layer.

The algorithm implementation layer is the actual core part of the framework because it takes on the role of creating test data. On the other hand, the algorithm support layer takes on the role of defining auxiliary tools required to create test data. In the test case generation layer, test case generation algorithms are implemented using data structures. An executable model and a model data structure are generated in the model abstraction layer. The generated executable model is loaded by the model abstraction layer. Then, the executive control is moved to the test case generation layer, where the test case is generated. The test case generation algorithms can be easily implemented by using various functions, which is provided in the algorithm support layer.

In general, the state-based model is defined as 4-tuple *M* = (*S*; Π; *V*; *T*). *S*, Π, *V*, and *T* denote a set of states, a set of events, a set of variables, and a set of transitions, respectively [32]. The test input is defined as *I* = (*V*, Θ(*V*)), where *V* is a set of variables and Θ is values of *V*. For example, Θ(*m*) = 1 when *m* is 1. Therefore, the test input sequence Σ = {*I*_*x*_∣*x* = 1,…, *n*}, where *I*_1_ is the set of the test input on the initial step, and *I*_*n*_ is the set on the* n*th step. The set of Σ is the final goal to obtain because the test for a model can be composed of several input sequences.

The random algorithm is the easiest algorithm to be implemented. The algorithm selects an arbitrary candidate test input *I* among the candidates and determines whether or not to put *I* into the test input sequence after executing the executable model and analyzing the feedback from the executable model. If the algorithm decided that *I* would be a useful test input to cover the target coverage, then *I* is included in Σ. All steps of the test input should be randomly chosen. The same process iterates along each coverage goal of the target model.  Algorithm 1 shows the pseudocode of a random algorithm.

The candidate input is useful only if it can satisfy the coverage. The random algorithm is not able to provide any information about how to modify the candidate input to satisfy the coverage but is able to say whether or not the candidate input satisfies the coverage. Therefore, the random algorithm is not efficient in finding out the test input, though it is a quite simple and widely used algorithm as a benchmark target.

The local search algorithm or the metaheuristic search algorithm can also be implemented. For the algorithms, the representation and fitness functions of search targets should be defined. For treating the state-based model, the representation to describe the status of the state should be employed. Therefore, a represented node should have the status of the current model and the input sequence which has inputs from the initial state to the current state. The base node is used for the metaheuristic search algorithms as well. The traditional branch distance or the approach level is employed as the fitness function. The branch distance or the approach level can be included in the feedback, which is given by the executable model at the design level. A strong point of the methods is that they provide accurate values for the fitness function that can be obtained by computing the values dynamically.

Algorithm 2 shows pseudocodes of the discrete space hill climbing search method, which is one of local search methods [33]. We employed the algorithm for the discrete space because the fitness function may have discrete values of true/false not by continuous values but by Boolean values in the state-based model. A start node is a start position from which the searching for the target coverage begins. For example, to satisfy the transition coverage, the algorithm chooses a node as a start node that includes the source state of the target transition. The algorithm generates 100 neighbor nodes of the current node and compares the current node and the best neighbor which has the highest fitness among the generated neighbor nodes. If the fitness of the best neighbor is higher than the current node, then the best neighbor becomes the current node. The algorithm iterates until it cannot find out any neighbor which has higher fitness than the current node. As known already, the local search suffers from the local optimum or plateau problems. The local search algorithm works very effectively where local optimum or plateau does not exist.

Genetic algorithms (GA) are one of the most studied research algorithms on the metaheuristic search. In GA, each individual in a population evolves from one generation to more developed generations by following the selection, crossover, and mutation stages. Various algorithms have been presented in accordance with the type of operations. For selections, linear ranking-based selections and tournament-based selection are presented. Examples of the linear ranking-based algorithm are roulette wheel selection, stochastic universal sampling, and trunk ranking selection. Examples of the tournament-based selection are 2-way tournament, 3-way tournament, or *k*-way tournament selections, each of which compares 2 individuals, 3 individuals, and *k* individuals, respectively. We can employ a general GA algorithm to MBT because the selection algorithm selects better individuals using the fitness function.  Algorithm 3 shows the pseudocode of the genetic algorithm.

The crossover operation is dependent on the representation of individuals. An individual has a chromosome, which consists of several genes. Crossover operation is mixing up genes. An individual has the status of a current model and the input sequence from the initial state to the current state. An input sequence is considered as a chromosome, and one step is mapped to a gene of a chromosome. The crossover is the operation crossing each step. Single point crossover is different from multipoint crossover in that it has only one crossover point. Uniform crossover, which selects a gene from parents at each point, and cut-and-splice crossover, which crosses over the steps at the different points of parents, can be implemented in this framework.

The mutation operation mutates an individual to solve the local optima or plateau problems. GA changes the values of genes. Because a gene is mapped to an input step of MBT, we can use the method of changing the value in a step. The method of erasing or adding one step can be also used.

#### 3.2.2. The Algorithm Support Layer

The algorithm support layer covers the common tool modules which can be used with any algorithm. Just like libraries, this part can be commonly used on various models. By reusing these common modules, the development time can be shortened and the program is getting more reliable. Essential functions which should be provided by the algorithm support layer are coverage goal generation, executable model control, and feedback analysis. Whereas traditional design pattern techniques present at most forms of classes, the proposed framework also provides useful functions for the generation of test cases.

The coverage gives directions for test case generation. State coverage and transition coverage are widely used for state-based models. MC/DC (MC/DC is one of the standard coverage factors required for DO-178B/C in the aerospace domain and ISO 26262 in the automotive domain.) (modified condition/decision coverage) may be required for the safety-critical software testing. One of the most important performance matrices for a test case generator is how much the coverage goal is satisfied by the generated test case. The generated test cases should visit all states of the model for the state coverage and all transitions of the model for the transition coverage. MC/DC is for the test to see how each condition (event and guard condition) in every transition would independently affect the condition and decision.

The state coverage and transition coverage create a goal as a list of all states and transitions included in the model, respectively. The way to create the goal for MC/DC is that we regard each condition in every transition as a predicate of a branch condition.

The proposed framework provides the common functions in the algorithm support layer to control the executable model generated in the model abstraction layer. The functions should support model initialization, model execution, model termination, candidate test input setting, execution results acquisition, and model status acquisition as mentioned earlier.

The feedback of the executable model shows how the candidate test input fits to the coverage goal. After executing the model with the candidate test inputs, the executable model should provide the visited transitions and the branch distance values of the transition so that the test case generator may calculate the fitness value. Based on the outputs of the executable model, the generated test cases should be mapped to the predefined coverage goal in order to check the coverage with the test cases. Moreover, the software framework should provide a function for calculating fitness values to be used in the metaheuristic search algorithm.

## 4. Case Studies

The primary purpose of the proposed framework is to give easy deployment of test case generator for the various types of models and algorithms by reusing as many common modules as possible. To show the effectiveness of the framework, test cases for the EFSM model and the SL/SF model are generated by using a random algorithm and a genetic algorithm. EFSM is one of the most studied models, and SL/SF is the most popular model used in the automotive and aerospace industries. Generally, genetic algorithms and local searches have been widely studied in SBST, and the random algorithm is also popularly used as a benchmark. We set SL/SF as the default type of a model and the genetic algorithm as the default algorithm. That is to say, we use a genetic algorithm while varying the types of models and an SL/SF model while varying algorithms. We implemented the test case generation program for each case based on the framework.

### 4.1. Case I: Changing Models (EFSM versus SL/SF)

**Figure 2:**
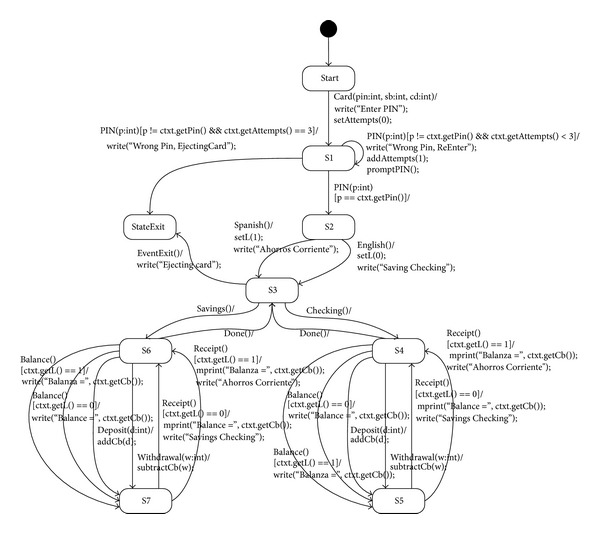
Sample of EFSM model: automatic teller machine.

The example of the EFSM model is an ATM model shown in Figure 2. GA is employed as the test case generation algorithm. The target model is generated with C code using a state machine compiler (SMC), as described in Section 3.1. Interfaces are added to the generated C code, and an executable model is generated from the source code and the interfaces. The generated test cases satisfy the transition coverage. Predefined files are additionally used because events and data are not defined in the input files. By using the predefined files, the total event number and maximum/minimum of parameter values can be obtained.

**Figure 3:**
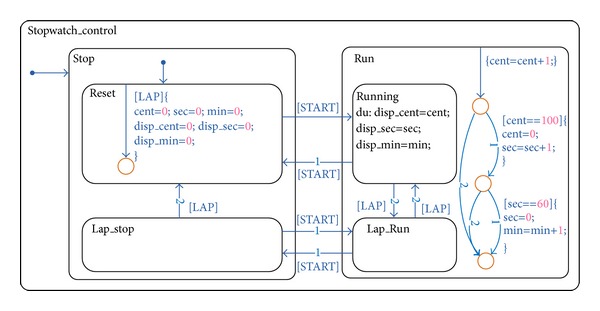
Sample of SL/SF model: stopwatch.

We assessed experiments by using the example shown in Figure 3 for the SL/SF model. The presented example is widely used as a hard example for analytical methods to find out test inputs because the example includes a nested loop. The identical GA algorithm used for EFSM is also used for an SL/SF model. The target model is converted into C codes, and the executable model is generated from the codes and the interfaces. The test case generation layer runs by using the generated executable model, and the test cases satisfying the transition coverage are generated. Without additional files, because the whole information can be obtained from the SL/SF model, we can set the maximum and minimum on data for the SL/SF model.

Table 1 shows the LOC (line of code) of each layer of the program after implementing the test case generator for the EFSM model and the SL/SF model. We counted the number of executed lines of the code. The development efforts are estimated as person-months (PM) using COCOMO II (http://csse.usc.edu/csse/research/COCOMOII/cocomo_main.html). We employ LOC for counting the number of lines, while comments and blank lines are excluded. It took 939 LOC for EFSM and 1466 LOC for SL/SF to convert the model into the executable model and the data structure into the structure, respectively. SL/SF model requires more source codes for the model abstraction layer than the other model because SL/SF has more complicate model structure. The ratios of the whole source code are about 40% and 50%, respectively. Therefore, we can reduce the time to market by about 50% if we employ the framework presented in this paper to build a test case generator for a new model. Moreover, the time to market will be shortened much more if a parser for the target model exists because most parts of the model abstraction layer are codes for parsing the model files by using lex and yacc.

Equation (3) is the COCOMO II equation [34]:

(3) $$\begin{array}{c} \text{PM}=A\times \text{Siz}{\text{e}}^{E}\times \overset{n}{\underset{i=1}{\prod }}\text{E}{\text{M}}_{i}. \end{array}$$

The exponent *E* in (3) is an aggregation of five scale factors (SF) that account for the relative economies or diseconomies of scale encountered for software projects of different sizes [35]. The EM in (3) is the effort multipliers to reflect the software project environment characteristics. All scale factors and effort multipliers are assumed as a nominal value.* Size *is the size of software development, which is in units of thousands of source lines of code (KSLOC). *A* is the constant which is used to capture the multiplicative effects on effort with projects of increasing size.* n* denotes the number of effort multipliers. Table 1 shows the estimated efforts of each model to implement each part of the proposed framework. EFSM model requires 6.8 PM, and SL/SF model requires 8.6 PM. If SL/SF model is implemented after implementing EFSM model, it takes only 4.5 PM to generate test cases because the test case generation layer is used. Now, 4.1 PM is saved. So, the proposed framework is efficient to implement a test case generator for various models.

Table 2 shows the results of test case generation with an identical GA for EFSM and SL/SF. It is meaningless to directly compare the results for EFSM with those of SL/SF because the two models are totally different. However, the implementation of test cases generator for both models using the proposed framework is successful. Therefore, the proposed framework is effective to implement a test case.

### 4.2. Case II: Changing Algorithms (Random versus Metaheuristics)

Three algorithms, such as a random algorithm, a hill climbing (HC) algorithm, and a genetic algorithm (GA), are implemented for an SL/SF model. Random, HC, and GA are the most famous search algorithms.  Table 3 shows LOCs of the source codes by varying the algorithm. For each result, the algorithm implementation layer possesses about 15% of the total code, which means that our framework works very efficiently even if the algorithm is changed. Hill climbing and genetic algorithms have larger common parts than other algorithms because they process the search by using the fitness function.

Table 3 shows the estimated efforts of each algorithm to implement each part of the proposed framework as well. Random algorithm requires 8.6 PM, HC requires 8.2 PM, and GA requires 8.6 PM. If GA is implemented after HC is implemented, it takes only 1.2 PM to generate test cases because the algorithm implementation layer only requires to be reimplemented. Now, 7.4 PM is saved. This is the evidence of the efficiency of the proposed framework to implement a test case generator with various algorithms.

Table 4 shows results of test case generation with three kinds of search-based algorithms for SL/SF. The implementation of a test case generator with the three algorithms using the proposed framework is successful. Therefore, the proposed framework is effective to implement a test case generator with various algorithms.

## 5. Conclusion

In this paper, we proposed a model-independent software framework, which is effective for changing of the types of models or the algorithms. With the proposed framework, we assessed experimental measurements by varying models (e.g., EFSM and SL/SF) and algorithms (random, HC, and GA). From the results of the measurement, the proposed framework is able to generate test cases and to reduce the development time. The proposed framework surpasses typical design patterns proposed by previous related works. Three search algorithms are employed easily using the proposed framework. For the future work, we have a plan to apply the proposed framework to other various models such as FSM, UML, and Markov model. More case studies will be carried out with more complex models. The proposed framework can be extended to the other areas of the Search-Based Software Engineering such as requirement analysis, software design, and software maintenance.

## Figures and Tables

**Algorithm 1:**
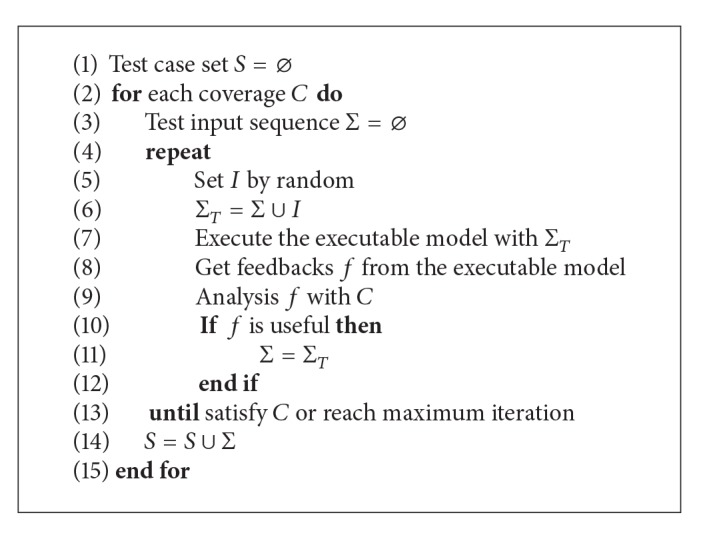
Pseudocode of random algorithm.

**Algorithm 2:**
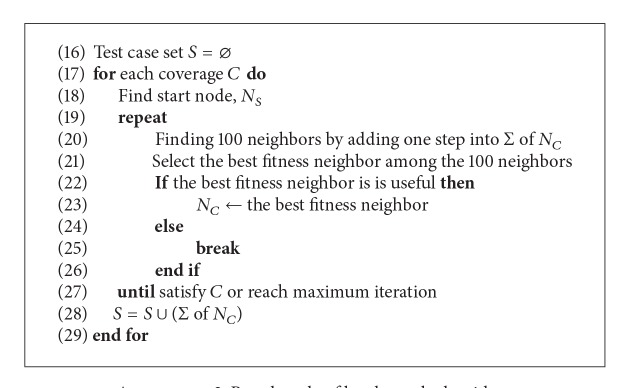
Pseudocode of local search algorithm.

**Algorithm 3:**
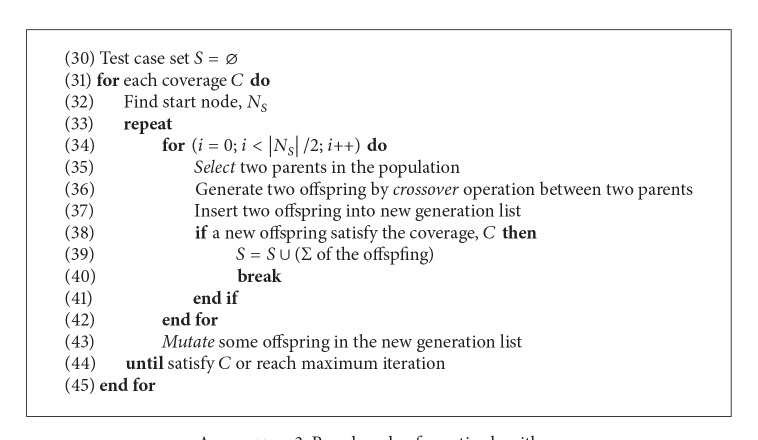
Pseudocode of genetic algorithm.

**Table 1:** Source code sizes of efforts of each model (using genetic algorithm).

	Model abstraction layer	Test case generation layer	
		Algorithm implementation layer	Algorithm support layer
EFSM	939 LOC (2.7 PM)	432 LOC (1.2 PM)	989 LOC (2.9 PM)
SL/SF	1466 LOC (4.5 PM)	432 LOC (1.2 PM)	991 LOC (2.9 PM)

**Table 2:** Test case generation results.

Models		Coverage	Number of test cases	Number of steps
EFSM	Max.	83%	15 ea	88 steps
	Min.	83%	11 ea	55 steps
	Ave.	**83%**	**13 ea**	**67.63 steps**

SL/SF	Max.	100%	10 ea	7052 steps
	Min.	93%	6 ea	131 steps
	Ave.	**97%**	**7.86 ea**	**3713.04 steps**

**Table 3:** Source code sizes and efforts of each algorithm (using SL/SF model).

Algorithm	Model abstraction layer	Test case generation layer	
		Algorithm implementation layer	Algorithm support layer
Random	1466 LOC (4.5 PM)	347 LOC (0.9 PM)	1066 LOC (3.2 PM)
HC	1466 LOC (4.5 PM)	299 LOC (0.8 PM)	991 LOC (2.9 PM)
GA	1466 LOC (4.5 PM)	432 LOC (1.2 PM)	991 LOC (2.9 PM)

**Table 4:** The result of test case generation.

Models		Coverage	Number of test cases	Number of steps
Random	Max.	80%	5 ea	54 steps
	Min.	73%	3 ea	20 steps
	Ave.	**78%**	**4.6 ea**	**35.67 steps**

HC	Max.	73%	4 ea	41 steps
	Min.	40%	1 ea	3 steps
	Ave.	**75%**	**2.06 ea**	**7.93 steps**

mGA	Max.	100%	10 ea	7052 steps
	Min.	93%	6 ea	131 steps
	Ave.	**96%**	**7.86 ea**	**3713.04 steps**
